# Carvedilol attenuates PTZ-induced epileptogenesis: associations with hippocampal neuroinflammation and PI3K/AKT/mTOR-related alterations

**DOI:** 10.1038/s41598-026-67306-2

**Published:** 2026-08-25

**Authors:** Ahmed H. Madkour, Rania R. Abdelaziz, Manar A. Nader, Mahmoud Elshal

**Affiliations:** 1 Department of Pharmacology and Toxicology, Faculty of Pharmacy, Mansoura University, Mansoura, 35516 Egypt; 2 Faculty of Health Science Technology, Mansoura National University, Gamasa, 7731168 Egypt

**Keywords:** Carvedilol, PTZ, NLRP3, PI3K/AKT/mTOR signaling, Diseases, Neurology, Neuroscience

## Abstract

Epileptogenesis is increasingly recognized as a multifactorial process driven by persistent neuroinflammation, oxidative stress, and dysregulated intracellular signaling, highlighting the need for therapeutic strategies that extend beyond symptomatic seizure control. The present study investigated the potential effects of prophylactic carvedilol administration on behavioral seizure progression and associated pathological alterations during the PTZ-induced kindling model, with particular emphasis on inflammatory and signaling pathways implicated in epileptogenesis. Repeated PTZ administration resulted in progressive behavioral seizure development, impaired open-field performance, hippocampal neuronal damage, reduced GABA levels, increased oxidative stress and inflammatory mediators, and altered hippocampal NLRP3 expression, along with changes in PI3K-, AKT-, and mTOR-related protein expression. Carvedilol treatment was associated with lower behavioral seizure scores, improved open-field performance, preservation of hippocampal architecture, restoration of GABA content, and attenuation of oxidative and inflammatory alterations. Carvedilol treatment was also accompanied by reduced NLRP3 levels and changes in PI3K/AKT/mTOR-related measurements. These findings demonstrate associations between carvedilol treatment and multiple pathological processes during PTZ-induced kindling, but do not establish direct pathway-specific causality.

**Supplementary Information:**

The online version contains supplementary material available at 10.1038/s41598-026-67306-2.

## Introduction

Epilepsy is a chronic neurological disorder characterized by recurrent, unprovoked seizures resulting from abnormal, excessive, and synchronous neuronal activity in the brain^1,2^. Epileptogenesis refers to the process by which a normal brain becomes susceptible to seizures over time, involving complex molecular, cellular, and network changes^3,4^. Regardless of considerable progress in antiseizure treatments, nearly one-third of patients still suffer from seizures despite proper pharmacological medications, emphasizing the demand for additional strategies targeting processes associated with seizure development and progression^5,6^.

Earlier studies revealed that neuroinflammation is a key pathway leading to epileptogenesis and seizure development^7^. Numerous studies show that seizure activity generates a wide inflammatory reaction in the brain comprising glial cells and neurons, specifically microglia and astrocytes, which dynamically contribute to the modelling of the epileptic network, contributing to long-term modifications in neuronal excitability and endorsing the development of repeated seizures^7,8^.

Nuclear factor kappa B (NF-κB) is one of the central mechanisms implicated in controlling the transcription of the inflammatory cascade in the brain^9^. Stimulation of NF-κB signaling causes upregulation of numerous pro-inflammatory cytokines such as tumor necrosis factor-alpha (TNF-α) and interleukin-1 beta (IL-1β), that displayed enhanced synaptic excitability and lower seizure threshold^7,10^. Collectively, these inflammatory reactions provide a self-sustaining neuroinflammatory loop that promotes epileptogenesis and seizure recurrence. NF-κB signaling also acts as a priming phase in nucleotide-binding oligomerization domain-like receptor protein 3 (NLRP3) inflammasome stimulation via the initiation of NLRP3 and pro-IL-1β expression, thus relating transcriptional inflammatory reactions to innate immune stimulation in epilepsy^11^. Triggering of the NLRP3 inflammasome consequently endorses caspase-1-dependent development of IL-1β and IL-18 activation, causing more amplification of neuroinflammatory signaling, neuronal damage, and epileptogenesis^12^.

Autonomously, oxidative stress is another crucial pathway in seizure-provoked neuronal damage, where extreme generation of reactive oxygen species (ROS) causes lipid peroxidation, mitochondrial dysfunction, and neuronal loss, especially in susceptible brain areas such as the hippocampus^13,14^. Oxidative stress and neuroinflammation are strongly interrelated pathways that intensify each other, starting a vicious cycle that aggravates neuronal injury and epileptogenesis^15,16^.

Beyond inflammatory and oxidative mechanisms, dysregulation of the phosphatidylinositol 3-kinase/protein kinase B/mechanistic target of rapamycin (PI3K/AKT/mTOR) signaling pathway has been increasingly implicated in epileptogenesis. This pathway regulates neuronal survival, synaptic plasticity, autophagy, and metabolic homeostasis, all of which are essential for maintaining neuronal network stability^17,18^. Emerging evidence indicates that the PI3K/AKT/mTOR pathway interacts closely with inflammatory signaling networks and contributes to the regulation of immune responses, neuronal survival, and synaptic plasticity^17,19^. Collectively, these interconnected NF-κB/NLRP3, oxidative stress, and PI3K/AKT/mTOR pathways form an integrated pathogenic network underlying epileptogenesis.

Given the multifactorial nature of epilepsy, drug repurposing has gained considerable attention as a promising strategy for identifying agents with multi-target neuroprotective properties. Carvedilol (CAR), a non-selective β-adrenergic receptor blocker widely used in cardiovascular diseases, has been reported to possess antioxidant and anti-inflammatory properties beyond its cardiovascular actions. It has demonstrated the ability to scavenge free radicals, reduce lipid peroxidation, and attenuate inflammatory responses in experimental models^20,21^. Moreover, its lipophilic nature suggests the potential for central nervous system penetration, as lipophilic β-adrenoceptor antagonists are known to readily cross the blood–brain barrier^22,23^. Notably, recent studies have shown that CAR may exert an anti-inflammatory effect^24,25^. A recent in-vivo study demonstrated that CAR pretreatment can significantly decrease serum TNF-α levels and nitrite levels by inhibiting NF-κB^26^.

Although the antioxidant and anti-inflammatory properties of CAR have been reported in several experimental neurological and systemic injury models, its effects have not been comprehensively characterized in the pentylenetetrazole (PTZ) window-kindling paradigm. The present study therefore evaluated whether prophylactic CAR treatment was associated with concurrent changes in behavioral seizure progression, open-field performance, hippocampal histopathology, gamma-aminobutyric acid (GABA) content, oxidative status, inflammatory mediators, and PI3K/AKT/mTOR-related measurements. This study was designed as an integrated preclinical characterization rather than as a pathway-specific mechanistic investigation.

## Materials and methods

### Experimental animals

Adult male Sprague-Dawley (SD) rats weighing between 220 and 240 g were purchased from VACSERA (Agouza, Giza, Egypt). The rats were housed for two weeks with a 12 h light/dark cycle and unlimited access to food and water, *ad libitum*. This study received approval from Mansoura University Animal Care and Use Committee and the Faculty of Pharmacy’s Scientific Research Ethics Committee (code number: MU-ACUC (PHARM.PhD.23.01.9)). All animal experiments were conducted in accordance with the Guide for the Care and Use of Laboratory Animals (National Research Council, 8th edition, USA, 2011). This study was carried out in compliance with the ARRIVE guidelines.

### Drugs and chemicals

PTZ was purchased from Sigma-Aldrich Co., USA. Sodium Valproate (VAL) was obtained as Depakine^®^ (Sanofi, France) and administered intraperitoneally (I.P.). CAR was administered as Carvid^®^ tablets (25 mg; Apex Pharma, Egypt), which were finely crushed and suspended in 0.5% carboxymethylcellulose (CMC) immediately before administration.

### Experimental protocol

A modified PTZ-kindling protocol, known as the window (win)-PTZ method, was employed to induce chronic epilepsy. Rats received four PTZ (37.5 mg/kg, I.P.) every other day, followed by a 22-day PTZ-free window, then 3 final PTZ doses were given on 29, 31, and 33 days to finalize the kindling procedure. This process needs less PTZ doses, is considered less invasive, and establishes a fully kindled epileptic state compared to conventional PTZ-kindling models, while still reliably^27^.

**Fig. 1:**
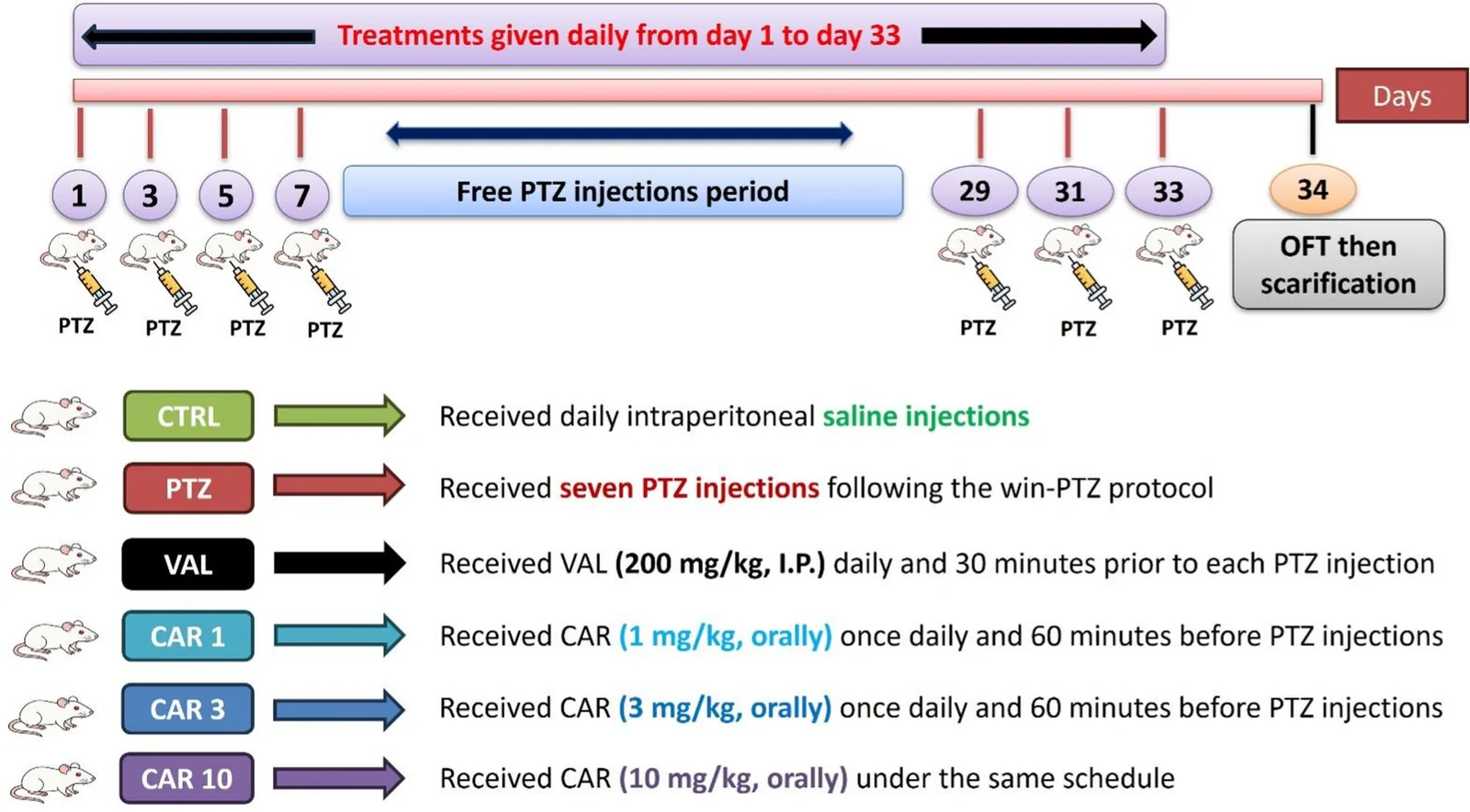
Schematic presentation of the experimental design.

Animals were arbitrarily allocated to six experimental groups (*n* = 6 per group) (Fig. 1**).** The sample size used in the present study was determined before initiation of the experiment using the resource equation approach, which is widely recommended for exploratory animal studies^28,29^:

- **Group 1** (CTRL): Received daily I.P. saline (PTZ vehicle) and oral CMC (drug vehicle).
- **Group 2** (PTZ): Received seven PTZ injections following the win-PTZ protocol and oral CMC.
- **Group 3** (VAL 200): Received VAL (200 mg/kg, I.P.) daily and 30 min prior to each PTZ injection^30^.
- **Group 4** (CAR 1): Received CAR (1 mg/kg, orally) once daily and 60 min before PTZ injections^31^.
- **Group 5** (CAR 3): Received CAR (3 mg/kg, orally) under the same schedule.
- **Group 6** (CAR 10): Received CAR (10 mg/kg, orally) under the same schedule^31^.

The selection of CAR doses (1, 3, and 10 mg/kg) was based on previously reported pharmacologically active dose ranges in rodent models rather than a single fixed dose. Previous studies have demonstrated biological effects of CAR at low doses (approximately 1–2 mg/kg), including attenuation of oxidative stress and improvement of behavioral deficits in neurodegenerative models^32^. In addition, higher doses within the range of 5–10 mg/kg have been investigated in experimental models of neurological injury and have shown antioxidant, anti-inflammatory, and neuroprotective effects^33,34^. Based on these reported active dose ranges, an intermediate dose of 3 mg/kg was incorporated to provide a graded dose-response design between low and high exposure levels. Therefore, the selected doses of 1, 3, and 10 mg/kg were intended to represent low, intermediate, and high pharmacologically active ranges, respectively, allowing assessment of CAR efficacy across increasing dose levels in the PTZ-kindling model.

Oral drug administration was performed using gastric gavage with an orogastric tube to ensure full delivery^35,36^. On day 34, behavioral assessments were carried out to evaluate locomotor function and cognitive performance.

The hippocampus was selected for all biochemical, molecular, and histopathological analyses because it is highly susceptible to seizure-induced injury and is one of the most extensively studied brain regions in experimental epilepsy^37^. Rats were deeply anesthetized with thiopental sodium (40 mg/kg, i.p.). After confirmation of an adequate depth of anesthesia, euthanasia was performed by cervical dislocation. The brains were immediately removed, rinsed in ice-cold saline, and carefully divided along the midline to separate the right and left hippocampal hemispheres. One hippocampal hemisphere was homogenized in 0.05 M phosphate buffer for enzyme-linked immunosorbent assay (ELISA) and Western blot analyses. Total protein concentration was determined according to the Bradford method^38^ using a commercial protein assay kit (Genei, Bangalore, India). The contralateral hippocampal hemisphere was sectioned and fixed in 10% neutral-buffered formalin for subsequent histopathological examination.

Accordingly, six rats were initially allocated to each experimental group. Importantly, no mortality occurred during the study; therefore, no animals were excluded from the experiment. Behavioral assessments and histopathological evaluation were performed using samples obtained from all animals. For molecular analyses, including Western blotting and selected pathway-specific measurements, fewer biological replicates were analyzed because these assays require substantial amounts of specialized reagents and are commonly performed on representative biological samples in experimental studies. The selected samples represented independent biological replicates from different animals and were not technical replicates. The exact sample sizes used for each experimental endpoint are specified in the corresponding assay sections and figure legends.

### Assessment of seizure score

Following each PTZ injection, animals were observed for 20 min to monitor convulsive behavior. Seizure severity was classified using the modified Racine scale^39^, as follows: Stage 0: No response, Stage 1: Ear and facial twitching, Stage 2: Convulsive waves through the body Stage 3: Myoclonic jerks and rearing, Stage 4: Clonic–tonic convulsions with the animal turning onto its side and Stage 5: Generalized clonic–tonic seizures accompanied by loss of postural control. Rats were considered fully kindled after exhibiting three consecutive seizures at stage 4 and/or 5 in response to the last three PTZ injections.

### Open Field Behavioral Test

The open field test (OFT) was employed to assess locomotor activity, exploratory behavior, and anxiety-like features commonly associated with central nervous system disorders^40^. The test apparatus consisted of a square open-field box (divided into 16 equal squares) enclosed by high opaque walls, as previously described by Valvassori and Varela^41^. Each rat was gently placed at the center of the arena, and behavior was recorded for 5 min; the following parameters were calculated:

- Latency to movement initiation (in seconds),
- The number of squares crossed (all four paws inside),
- Frequency of rearing (vertical exploratory activity),

Rats were adapted to the experimental room preceding the examination to reduce stress and variability. The field was fully cleansed with 70% ethanol between trials and groups to remove remaining aromas^42^.

### Hippocampal histopathological examination

The right portion of the hippocampus was set in 10% buffered formalin for 48 h, then cautiously dissected and embedded in paraffin. Serial coronal slices (4–5 μm thick) were made via a rotary microtome. Slices were stained with hematoxylin and eosin (H&E) to assess cellular morphology, neuronal integrity, and tissue architecture. Other sections were stained with cresyl violet (Nissl stain) to examine neuronal density and morphology in hippocampal subfields, especially in cornu ammonis area 3 (CA3), highlighting Nissl bodies in neuronal cytoplasm, with obvious visualization of viable neurons against degenerated ones. Stained sections were examined under a light microscope (Olympus Corporation, Tokyo, Japan) by a blinded pathologist. Histopathological changes were further evaluated using a multiparametric semiquantitative scoring system adapted from Tomita et al.^43^, with minor modifications. Two parameters were independently assessed: neuronal degeneration/necrosis and apical dendritic loss. Each parameter was graded on a scale of 0–3 (0 = absent, 1 = mild, 2 = moderate, and 3 = severe). The individual scores were summed to generate a cumulative histopathological score for each animal. The cumulative histopathological score was used for statistical analysis. Histopathological scoring was performed for all animals (*n* = 6/group) by a blinded pathologist. Representative photomicrographs from one animal per group are presented in the corresponding figures.

### Determination of ELISA biomarkers

Hippocampal tissue homogenates were used for the determination of GABA; (Cat. No. ER1707, FineTest, Wuhan, Hubei, China), malondialdehyde (MDA); (Cat. No. MBS268427, MyBioSource, Inc., San Diego, CA, USA), total antioxidant capacity (TAC); (Cat. No. AMS.E02T0028, AMSBIO, Cambridge, UK), NLRP3 (Cat. No. MBS2033695, MyBioSource, Inc., San Diego, CA, USA), TNF-α (Cat. No. 438204, BioLegend, San Diego, CA, USA), NF-κB p65 (Cat. No. CSB-E08788r, CUSABIO Technology Co., Ltd., Wuhan, Hubei, China), IL-1β (Cat. No. RLB00, R&D Systems, Inc., Minneapolis, MN, USA), PI3K (Cat. No. ER1910, FineTest, Wuhan, Hubei, China), and mTOR (Cat. No. ER1520, FineTest, Wuhan, Hubei, China) using commercially available assay kits according to the manufacturers’ instructions. Standard calibration curves were generated using the supplied standards for each assay, and all standards and tissue homogenate samples were analyzed in duplicate. Optical density was measured at 450 nm using an automated microplate reader, with reference wavelength correction where recommended by the manufacturer. Analyte concentrations were calculated from the corresponding standard curves using a four-parameter logistic regression model according to the manufacturer’s instructions. The assay characteristics, including assay format, standard calibration concentrations, detection ranges, sensitivities, and absorbance wavelengths, are summarized in **Supplementary Table ****S1**.

### Assessment of hippocampal AKT by western blot

Hippocampal tissue was homogenized, and total protein was extracted using the ReadyPrep™ Protein Extraction Kit (Bio-Rad, Cat. #163–2086) according to the manufacturer’s instructions. Protein concentration was quantified using the Bradford Protein Assay Kit (Bio Basic, Cat. #SK3041). Equal protein amounts (20 µg) were denatured in 2× Laemmli buffer (containing 4% Sodium Dodecyl Sulfate (SDS), 10% 2-mercaptoethanol, 20% glycerol, 0.004% bromophenol blue, and 0.125 M Tris-HCl, pH 6.8) by heating at 95 °C for 5 min.

Proteins were separated via SDS-polyacrylamide gel electrophoresis and transferred to polyvinylidene difluoride membranes. Membranes were blocked in 3% bovine serum albumin in tris-buffered saline with Tween-20 (0.1% Tween-20) for 1 h at room temperature and incubated overnight at 4 °C with primary antibodies against total AKT Antibody (#9272), and Phospho-AKT (#sc-293125) from Cell Signaling Technology (Danvers, MA, USA) and Santa Cruz Biotechnology, Inc. (Dallas, TX, USA), respectively. After washing, membranes were incubated for 1 h at room temperature with appropriate horseradish peroxidase-conjugated secondary antibodies (Novus Biologicals).

Detection was performed using an enhanced chemiluminescence substrate (Bio-Rad, Cat. #170–5060), and signals were visualized using a ChemiDoc™ MP Imaging System (Bio-Rad, USA). Band intensities were quantified using image analysis software. The intensity of each target protein band was normalized to β-actin (#4967) from Cell Signaling Technology (Danvers, MA, USA) as an internal loading control. The relative phosphorylation status of AKT was calculated as the ratio of phospho-AKT expression to total AKT expression (p-AKT/AKT ratio), which was used for statistical analysis. Western blot analysis was performed using three independent hippocampal samples from each group. Each sample represented a distinct biological replicate derived from an individual animal.

### Statistical analyses

The sample size was determined a priori using the resource equation approach, which was considered appropriate for the present exploratory animal study. Each experimental group initially included six animals. Data were assessed for normality using the Shapiro–Wilk test. Parametric data are presented as mean ± standard deviation (SD), whereas non-parametric data are presented as median with interquartile range (IQR). One-way analysis of variance (ANOVA) followed by Tukey–Kramer post hoc test was used for parametric comparisons, whereas the Kruskal–Wallis test followed by Dunn’s multiple-comparison test was used for non-parametric comparisons. Seizure severity scores recorded repeatedly across the seven PTZ injections were analyzed using the Friedman test for repeated measures, followed by Dunn’s multiple-comparison test when significant. In addition, Kruskal–Wallis tests followed by Dunn’s multiple-comparison tests were performed separately at each PTZ injection to assess differences among the experimental groups at each time point. Statistical analyses were performed using GraphPad Prism version 10.1.0 (GraphPad Software, San Diego, CA, USA). The exact sample size used for each endpoint is indicated in the corresponding figure legend. A *p*-value < 0.05 was considered statistically significant. *P*-values were reported as provided by the statistical software; exact numerical values were reported where available, whereas values below the reporting threshold were presented as *p* < 0.0001.

## Results

### Effect of CAR and VAL on seizure severity in the PTZ-induced kindling model

As shown in Table 1, the CTRL group exhibited no seizure activity throughout the experimental period, maintaining a Racine score of 0 following all PTZ injections. In contrast, repeated PTZ administration induced a progressive increase in seizure severity, with seizure scores increasing from 1 ± 0.75 after the first injection to 5 ± 0 following the sixth and seventh injections, confirming the successful establishment of the PTZ-kindling model. Significant differences among groups were observed at each PTZ injection, with large effect sizes (ε² = 0.745–0.991) indicating a substantial magnitude of group differences throughout the kindling protocol.

**Table 1:** Effect of CAR and VAL on seizure severity in the PTZ-induced kindling model:.

Groups	Seizure severity scores after PTZ injections						
	First	Second	Third	Fourth	Fifth	Sixth	Seventh
CTRL	0 ± 0	0 ± 0	0 ± 0	0 ± 0	0 ± 0	0 ± 0	0 ± 0
PTZ	1 ± 0.75^*^	2 ± 0.75^*^	2 ± 0^*^	3 ± 0.75^*^	4.5 ± 1^*^	5 ± 0^*^	5 ± 0^*^
VAL	0 ± 0^#^	0 ± 0^#^	0 ± 0^#^	0.5 ± 1^#^	1 ± 0.75^#^	1 ± 0^#^	1 ± 0^#^
CAR 1	0 ± 0^#^	1 ± 0^*$^	1.5 ± 1^*$^	2 ± 0^*#$^	2.5 ± 1^*#$^	3 ± 0.75^*#$^	3 ± 0.75^*#$^
CAR 3	0 ± 0^#^	0 ± 0^#%^	1 ± 0.75^#^	1 ± 0.75^#%^	1.5 ± 1^*#%^	2 ± 0.75^*#%^	2 ± 0^*#%^
CAR 10	0 ± 0^#^	0 ± 0^#%^	0 ± 0 ^#%^	1 ± 0.75^#%^	1 ± 0 ^#%^	1 ± 0.75^#%^	1.5 ± 1^#%^
*p-*value	< 0.0001	< 0.0001	< 0.0001	< 0.0001	< 0.0001	< 0.0001	< 0.0001
ε²	0.991	0.966	0.809	0.847	0.745	0.853	0.895

Data were expressed as median ± IQR. Seizure severity scores across the seven PTZ injections were analyzed using the Friedman test for repeated measures followed by Dunn’s multiple-comparison post hoc test. Differences among experimental groups at each PTZ injection were analyzed using the Kruskal–Wallis test followed by Dunn’s multiple-comparison test (*n* = 6, *p*˂0.05). *p*-values and epsilon squared (ε²) effect sizes are provided for each PTZ injection.

* Significant Vs CTRL, # Significant Vs PTZ, $ Significant Vs VAL 200, % Significant Vs CAR 1 and & Significant Vs CAR 3 (*p*˂0.05).

VAL (200 mg/kg) administration markedly attenuated seizure progression throughout the study. Compared with PTZ-kindled rats, VAL-treated animals exhibited significantly lower seizure scores, which remained close to score 1 during the late stages of kindling. Similarly, CAR administration produced a dose-dependent reduction in seizure severity. CAR at 1 mg/kg reduced seizure scores compared with the PTZ group; however, its protective effect was less pronounced than that observed with VAL. CAR at 3 mg/kg produced greater attenuation of seizure progression, particularly during the advanced stages of kindling. The highest protective effect was observed with CAR 10 mg/kg, which maintained seizure scores between 0 and 1.5 throughout the protocol and markedly suppressed seizure progression compared with PTZ-kindled rats. Notably, the effect of CAR 10 mg/kg was comparable to VAL throughout the kindling protocol.

### Effect of CAR and VAL on OFT in win-PTZ-induced kindling model

Performance in the OFT was markedly impaired in PTZ-kindled rats compared with the CTRL group. PTZ administration significantly increased the latency to initiate movement while reducing both the number of squares crossed and rearing frequency, indicating diminished locomotor and exploratory activities. Treatment with VAL significantly improved all behavioral parameters relative to the PTZ group.

Similarly, CAR administration ameliorated PTZ-induced behavioral deficits in a dose-dependent manner. Rats treated with CAR 1 mg/kg exhibited a significant reduction in latency to move accompanied by an increase in locomotor and exploratory activities compared with PTZ-kindled animals. Further improvement was observed with CAR 3 mg/kg, whereas the greatest behavioral recovery was achieved in the CAR 10 mg/kg group. At this dose, latency to move was reduced to values approaching those of the CTRL group, while both the number of squares crossed and rearing events significantly increased compared with PTZ-treated rats. Notably, the behavioral performance of animals receiving CAR 10 mg/kg was comparable to that observed in the VAL-treated group for most measured parameters.

**Fig. 2:**
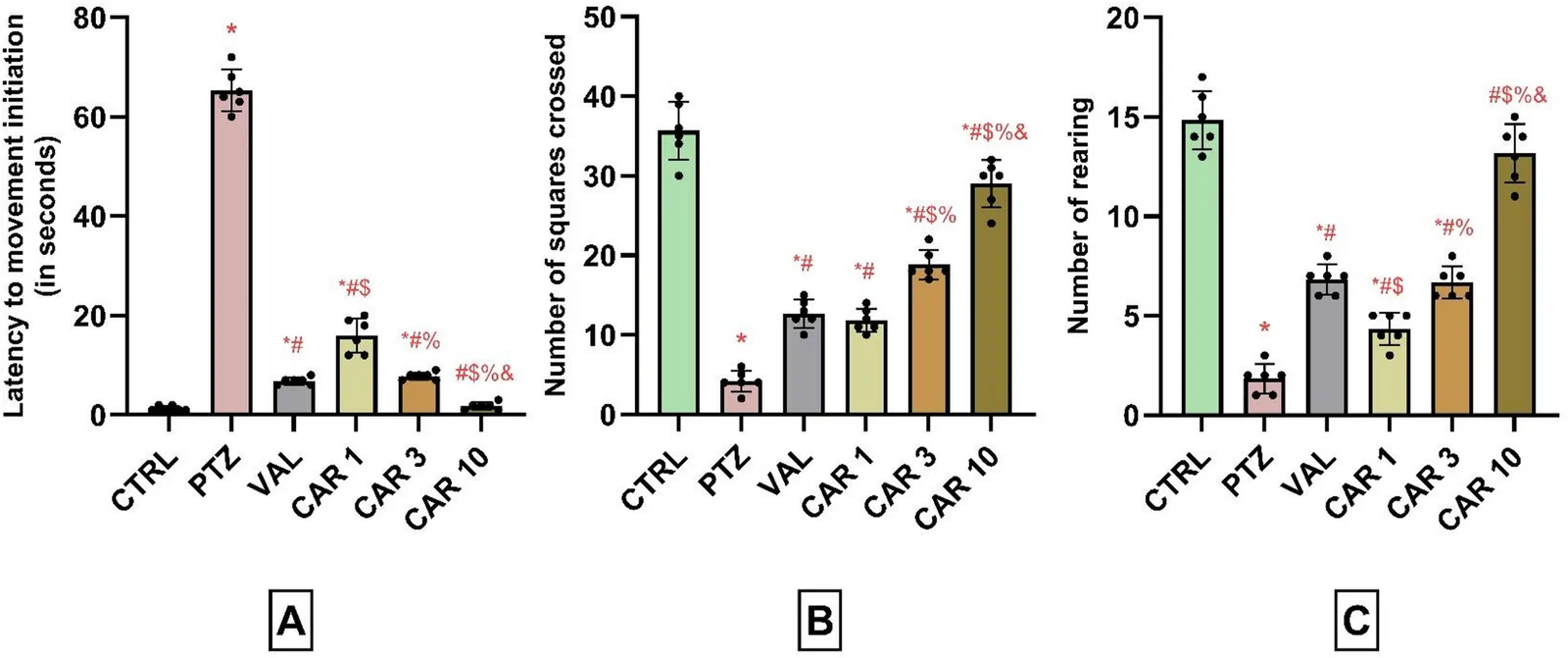
**Effect of CAR and VAL on OFT in win-PTZ-induced kindling model: (A); latency**,** (B); number of squares crossed**,** (C); Number of rearing.** Data are presented as mean ± SD. Statistical analysis was performed using one-way ANOVA followed by Tukey–Kramer’s multiple comparisons test (*n* = 6/group). *p*-values and effect sizes (η²) are reported. * Significant Vs CTRL, # Significant Vs PTZ, $ Significant Vs VAL 200, % Significant Vs CAR 1 and & Significant Vs CAR 3 (*p*˂0.05).

Collectively, these findings demonstrate that CAR effectively attenuated PTZ-induced impairments in locomotor and exploratory behavior, with the highest tested dose producing the most pronounced behavioral improvement. One-way ANOVA revealed significant differences among groups across all measured OFT parameters (**latency**: *p* < 0.0001, η² = 0.991; **number of squares crossed**: *p* < 0.0001, η² = 0.962; **number of rearings**: *p <* 0.0001, η² = 0.958), indicating a substantial magnitude of group differences in locomotor and exploratory behaviors **(**Fig. 2A-C**)**.

### Effect of CAR and VAL on hippocampal histopathological changes in PTZ-induced kindling model

(A) **H&E-stained sections**.

**Fig. 3:**
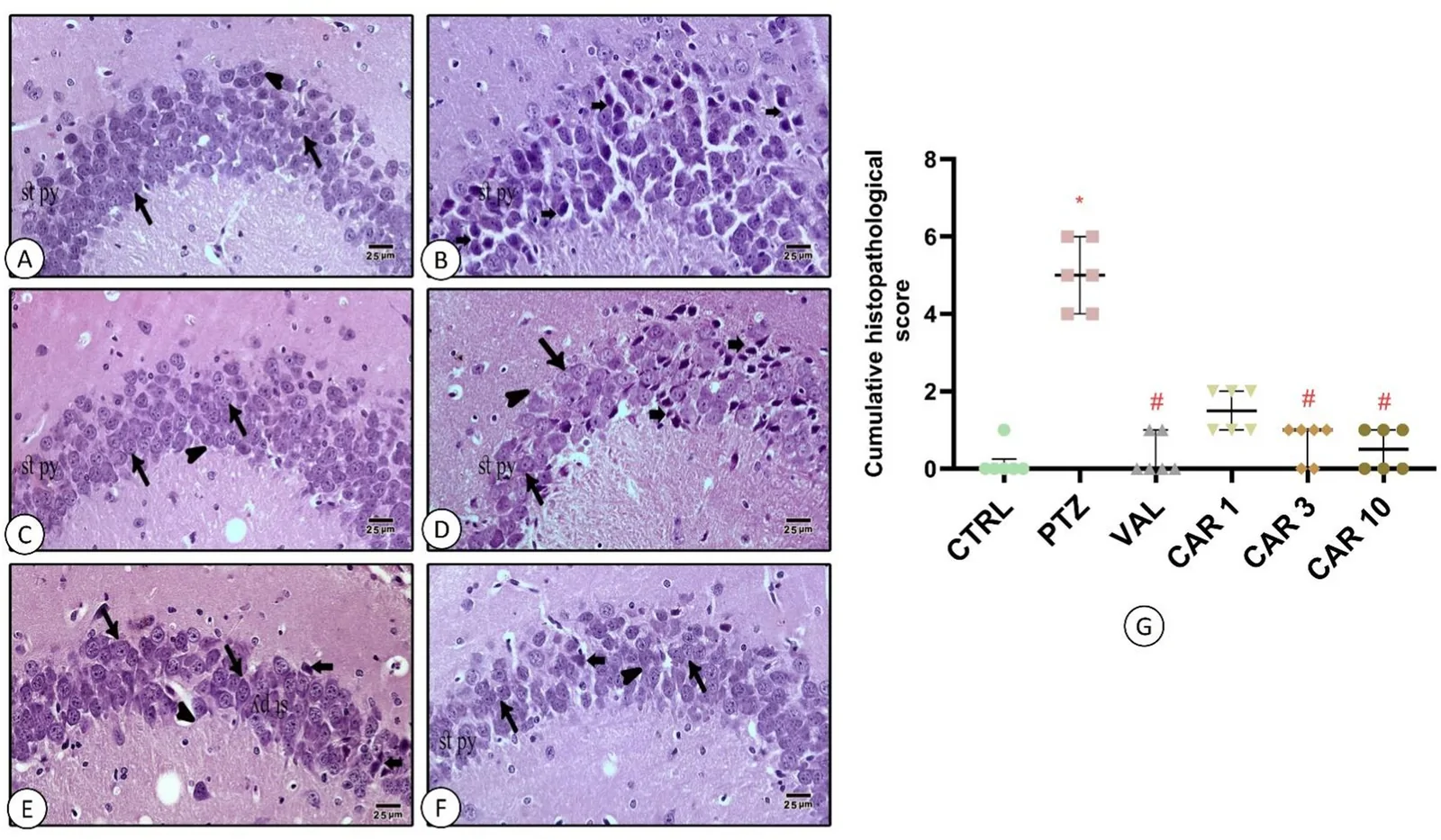
**Effect of CAR and VAL on hippocampal histopathological changes (H&E) in PTZ-induced kindling model: (A);** CTRL group, **(B);** PTZ group, **(C);** VAL 200 group, **(D);** CAR 1 group, **(E);** CAR 3 group, **(F);** CAR 10 group, (**G**); **Cumulative histopathological score. (st.py)**: stratum pyramidale, **(arrowhead)**: The apical dendrites, **(thick arrows)**: shrunken, darkly stained pyramidal cells surrounded by haloes of empty spaces, **(thin arrows)**: large-sized triangular pyramidal cells. magnification X:400. Data are presented as median ± interquartile range (IQR). Statistical analysis was performed using the Kruskal–Wallis test followed by Dunn’s multiple comparisons post-hoc test (*n* = 6/group). *p*-values and epsilon squared (ε²) effect size are reported. * Significant Vs CTRL, # Significant Vs PTZ (*p*˂0.05).

Photomicrographs of H&E-stained sections from the rat hippocampus revealed well-preserved neuronal architecture in the CTRL group. The CA3 region showed large triangular pyramidal cells with vesicular nuclei arranged in five to six loosely packed layers. The apical dendrites arose from the apex of the cells, whereas the basal dendrites arose from their basal portions **(**Fig. 3A**).** In the PTZ group, most pyramidal cells in the stratum pyramidale (st.py) appeared shrunken and darkly stained and were surrounded by halos of empty spaces. These cells showed loss of their apical dendrites **(**Fig. 3B**)**. Similar to the CTRL group, the VAL group exhibited a well-preserved stratum pyramidale composed of large triangular pyramidal cells with vesicular nuclei and conspicuous nucleoli arranged in five to six tightly packed layers **(**Fig. 3C**)**. In the CAR 1 group, some pyramidal cells appeared shrunken and darkly stained with loss of their apical dendrites. However, other pyramidal cells retained their triangular morphology with vesicular nuclei and prominent nucleoli **(**Fig. 3D**)**. The CAR 3 group showed fewer shrunken, darkly stained pyramidal cells together with a greater proportion of morphologically preserved neurons than the CAR 1 group **(**Fig. 3E**).** The CAR 10 group exhibited largely preserved neuronal morphology, with most pyramidal cells displaying vesicular nuclei and preserved apical dendrites. Only a few shrunken, darkly stained pyramidal cells with loss of their apical dendrites were observed **(**Fig. 3F**).**

Semiquantitative analysis demonstrated a significant increase in the cumulative histopathological score in the PTZ group compared with the CTRL group (adjusted *p* = 0.0005). Treatment with VAL significantly reduced the cumulative histopathological score compared with the PTZ group (adjusted *p* = 0.0028). CAR 3 and CAR 10 also significantly reduced the cumulative histopathological score compared with the PTZ group (adjusted *p* = 0.0467 and 0.0122, respectively). In contrast, CAR 1 did not significantly reduce the cumulative histopathological score (adjusted *p* > 0.9999) **(**Fig. 3G**).** The Kruskal–Wallis analysis revealed a significant difference in the cumulative histopathological score among groups (H = 24.68, *p* = 0.0002), with a large effect size (ε² = 0.656).

(B) **Nissl-stained sections**.

**Fig. 4:**
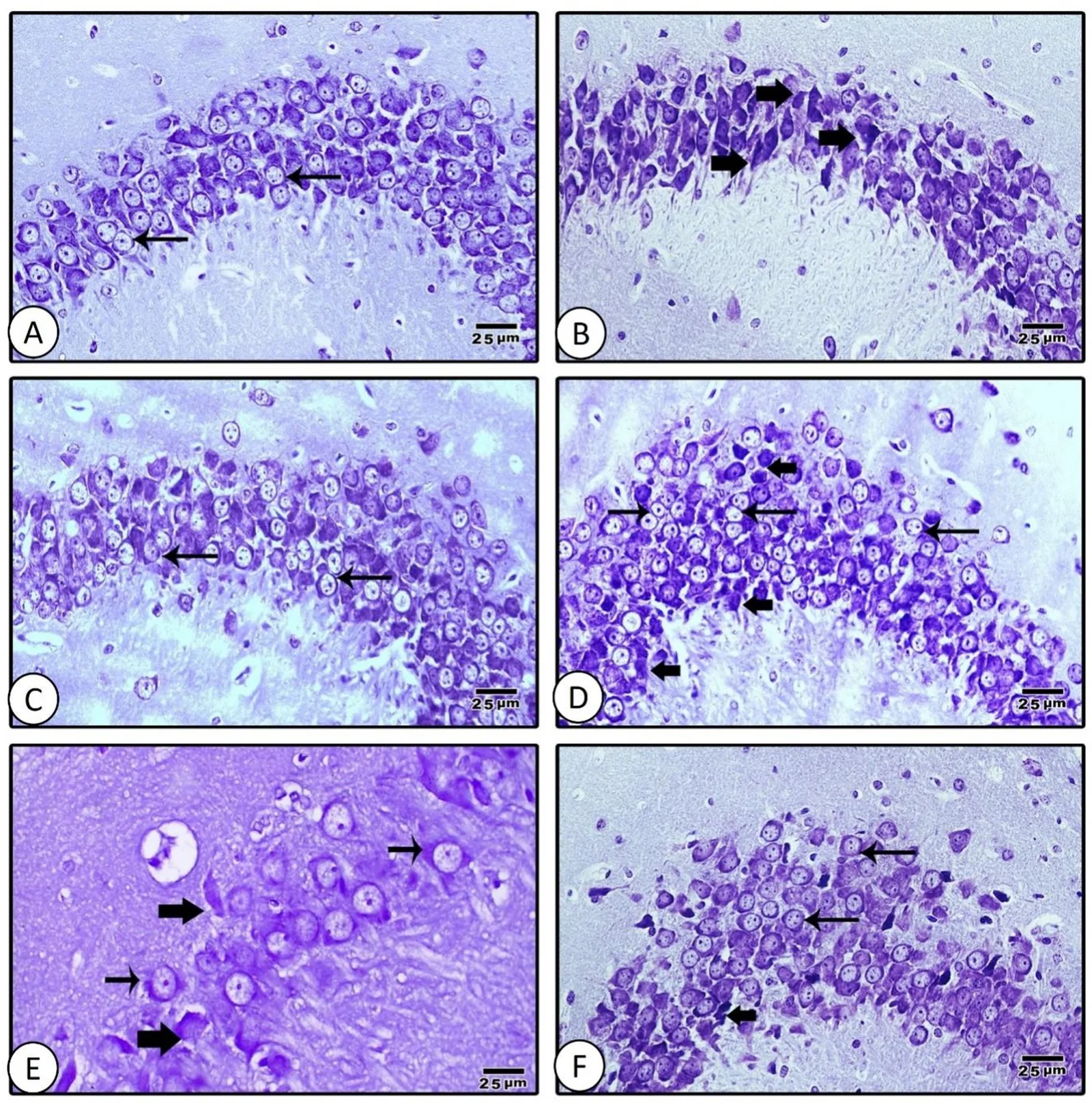
**Effect of CAR and VAL on hippocampal histopathological changes on hippocampal histopathological changes (Nissl-stained sections) in PTZ-induced kindling model: (A);** CTRL group, **(B);** PTZ group, **(C);** VAL 200 group, **(D);** CAR 1 group, **(E);** CAR 3 group, **(F);** CAR 10 group. **(thin arrow)**: small-sized rounded pyramidal cells with distinct boundaries and abundant Nissl substance; **(thick arrows)**: shrunken darkly stained pyramidal cells with indistinct boundaries and depleted Nissl substance; magnification X:400.

The Nissl-stained sections were consistent with the findings observed in the H&E-stained preparations. In the CTRL group, the CA3 region showed well-preserved neuronal architecture with abundant Nissl substance. In contrast, the PTZ group displayed extensive neuronal damage, characterized by shrunken, darkly stained neurons and marked loss of Nissl substance. Similar to the CTRL group, the VAL group showed largely preserved neuronal architecture and Nissl staining. CAR treatment produced dose-dependent preservation of neuronal morphology and Nissl staining. Among the CAR-treated groups, the CAR 10 group showed the greatest preservation of neuronal morphology and Nissl staining **(**Fig. 4**).**

### Effect of CAR and VAL on hippocampal levels of GABA in PTZ-induced kindling model

**Fig. 5:**
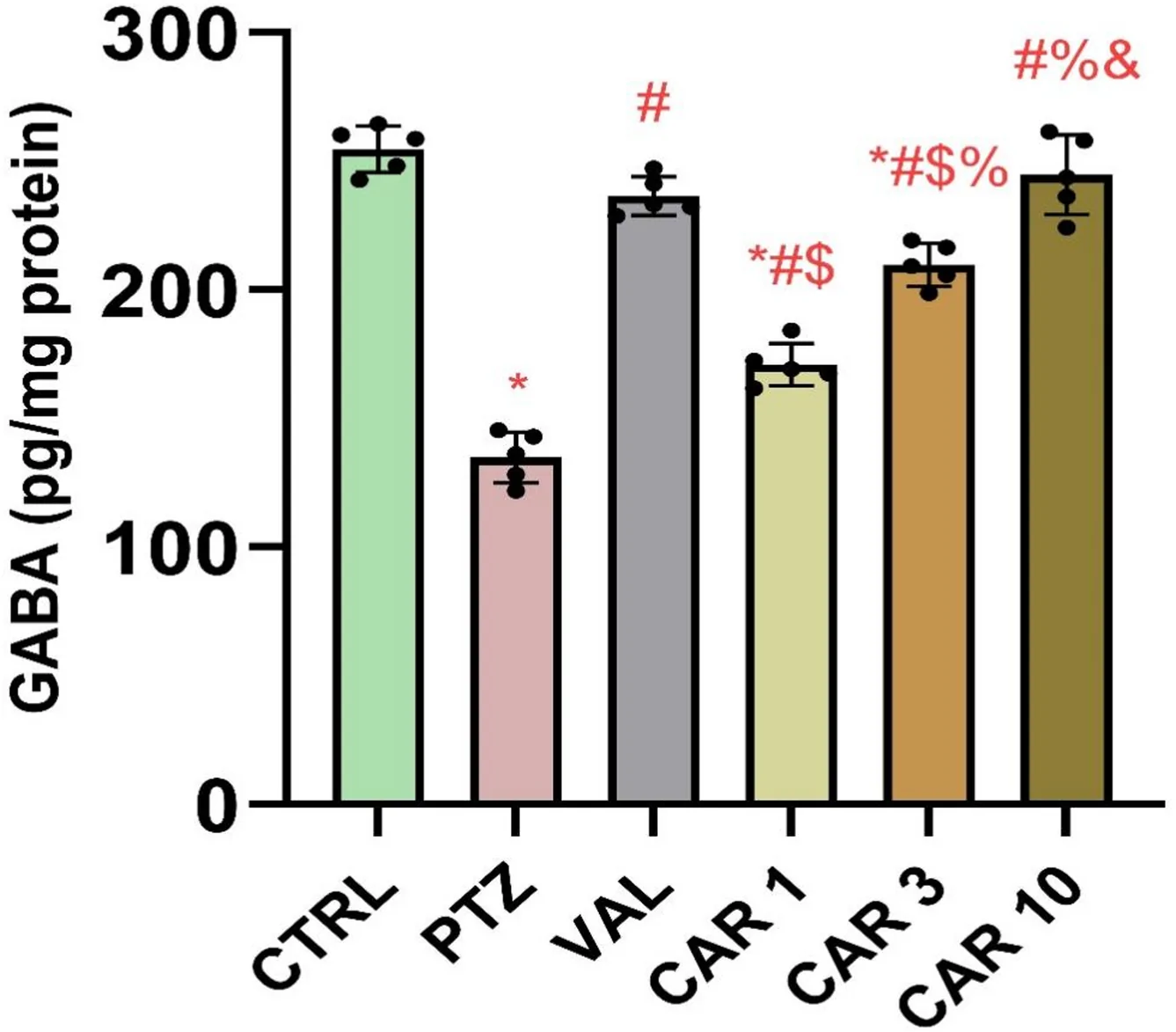
**Effect of CAR and VAL on hippocampal levels of GABA in PTZ-induced kindling model**: Data are presented as mean ± SD. Statistical analysis was performed using one-way ANOVA followed by Tukey–Kramer’s multiple comparisons test (*n* = 5/group). *p*-values and effect size (η²) are reported. * Significant Vs CTRL, # Significant Vs PTZ, $ Significant Vs VAL 200, % Significant Vs CAR 1 and & Significant Vs CAR 3 (*p*˂0.05).

PTZ-kindling markedly disrupted inhibitory neurotransmission, as evidenced by a significant reduction in hippocampal GABA levels, which declined by approximately 51% compared with the CTRL group. Treatment with VAL (200 mg/kg, i.p.) significantly restored GABA levels, resulting in an approximately 1.5-fold increase relative to the PTZ group. CAR administration dose-dependently upraised GABA levels. CAR at 1 mg/kg displayed a modest upsurge in GABA levels nearly 1.2-fold compared to the PTZ group, while CAR3 mg/kg administration generated a further noticeable restoration of GABA levels (nearly 1.4-fold increase) compared to the PTZ group. The highest CAR dose generated the maximum outcome, where GABA levels increased by nearly 1.9-fold compared to PTZ-kindled rats. The level of enhancement noticed with CAR 10 mg/kg was equivalent to that obtained with the VAL group. Overall, one-way ANOVA revealed significant differences among groups (*p* < 0.0001) with a large effect size (η² = 0.958), indicating a substantial magnitude of group differences in hippocampal GABA levels **(**Fig. 5**)**.

### Effect of CAR and VAL on hippocampal oxidant/antioxidant status in PTZ-induced kindling model

**Fig. 6:**
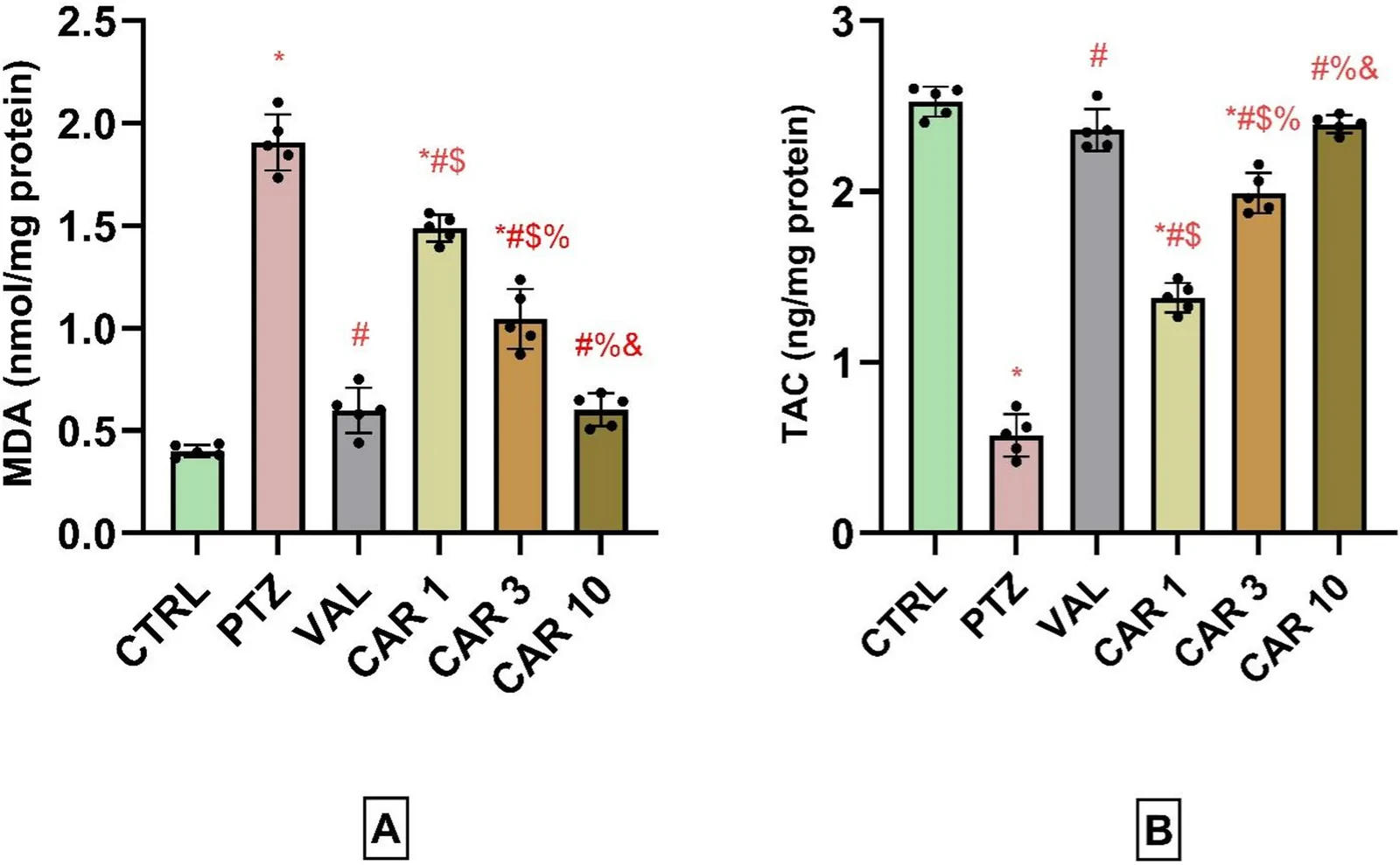
**Effect of CAR and VAL on hippocampal oxidant/antioxidant status in PTZ-induced kindling model: (A); MDA**,** and (B); TAC**. Data are presented as mean ± SD. Statistical analysis was performed using one-way ANOVA followed by Tukey–Kramer’s multiple comparisons test (*n* = 5/group). *p*-values and effect sizes (η²) are reported. * Significant Vs CTRL, # Significant Vs PTZ, $ Significant Vs VAL 200, % Significant Vs CAR 1 and & Significant Vs CAR 3 (*p*˂0.05).

Hippocampal oxidant/antioxidant balance was apparently disturbed in the PTZ group, as evinced by a 4.8-fold elevation in MDA levels and a 77.3% diminution in TAC compared to the CTRL group. VAL application considerably repaired redox homeostasis by dropping MDA levels (68.6%) and raising TAC (4.1-fold) compared to the PTZ group. CAR (1 mg/kg, orally) exhibited 22% MDA reduction and 2.4-fold elevation in TAC. CAR (3 mg/kg) disclosed a more pronounced effect, lowering MDA by 45.2% and raising TAC by 3.5-fold compared to the PTZ group. CAR (10 mg/kg) exhibited the greatest action, with a 68.4% decrease in MDA levels and a 4.1-fold increase in TAC compared to PTZ-kindled. One-way ANOVA revealed significant differences among groups for both MDA and TAC levels (*p* < 0.0001), with large effect sizes (η² = 0.972 for MDA and η² = 0.984 for TAC), indicating a substantial magnitude of group differences in hippocampal redox status **(**Fig. 6**)**.

### Impact of CAR and VAL on hippocampal NLRP3, TNF-α, NF-κB and IL-1β levels in PTZ kindled rats

**Fig. 7:**
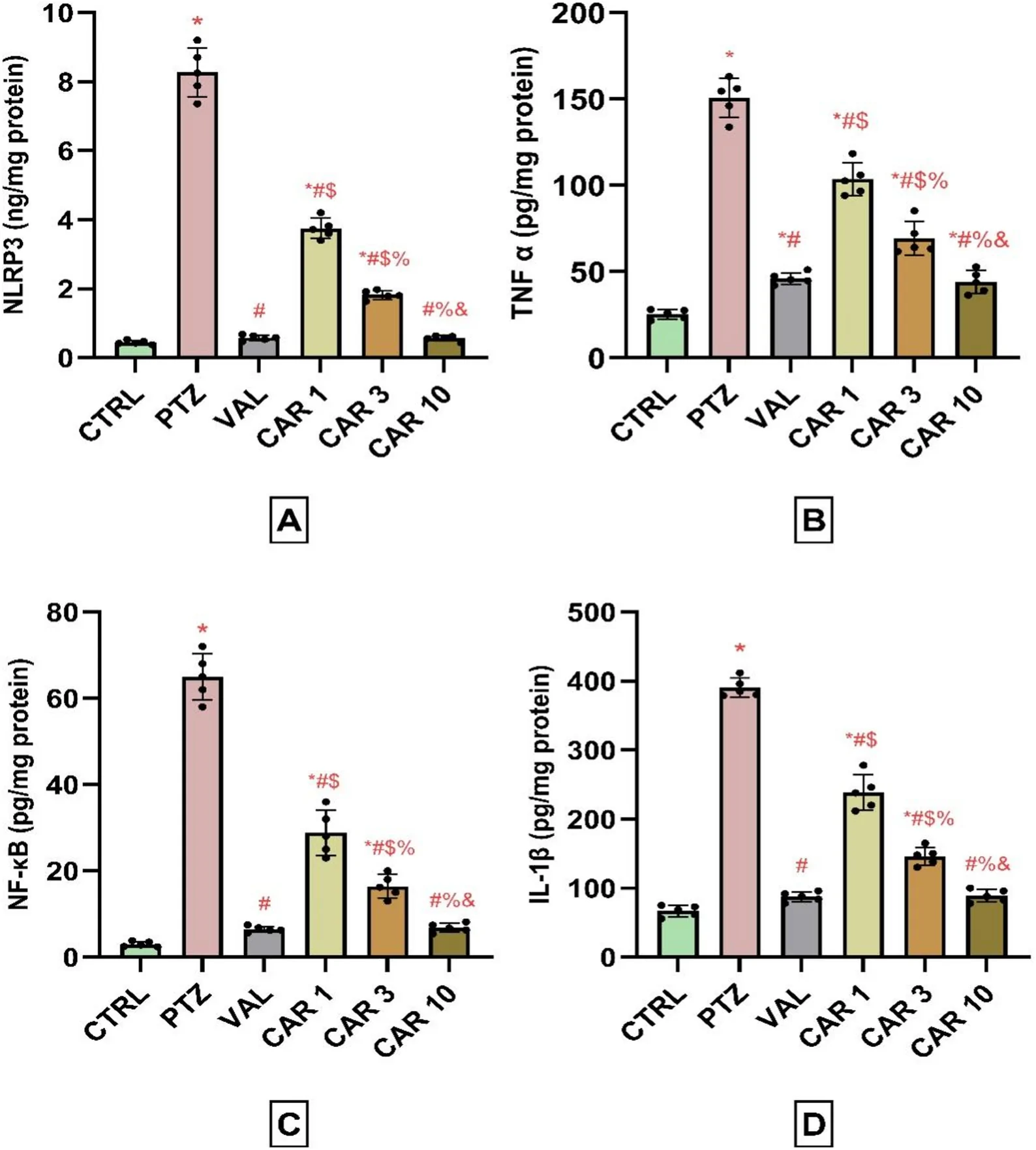
**Impact of CAR and VAL on hippocampal NLRP3**,** TNF-α**,** NF-κB and IL-1β levels in PTZ kindled rats: (A); NLRP3**,** (B); TNF-α**,** (C); NF-κB**,** and (D); IL-1β**: Data are presented as mean ± SD. Statistical analysis was performed using one-way ANOVA followed by Tukey–Kramer’s multiple comparisons test (*n* = 5/group). *p*-values and effect sizes (η²) are reported. * Significant Vs CTRL, # Significant Vs PTZ, $ Significant Vs VAL 200, % Significant Vs CAR 1 and & Significant Vs CAR 3 (*p*˂0.05).

PTZ-kindled rats exhibited substantially heightened hippocampal neuroinflammatory responses, as indicated by pronounced increases in NLRP3, TNF-α, NF-κB, and IL-1β levels compared to the CTRL group. PTZ administration increased hippocampal NLRP3, TNF-α, NF-κB and IL-1β levels by approximately 28-fold, 5.9-fold, 21-fold, and 5.8-fold, respectively. Treatment with VAL (200 mg/kg, i.p.) significantly attenuated these inflammatory alterations, reducing NLRP3, TNF-α, NF-κB, and IL-1β levels by approximately 92.8%, 69%, 90%, and 77%, respectively, compared with the PTZ group. Similarly, CAR treatment suppressed hippocampal neuroinflammation in a dose-dependent manner. Administration of CAR (1 mg/kg) reduced expression of NLRP3, TNF-α, NF-κB, and IL-1β by approximately 54%, 31%, 55%, and 38%, respectively, relative to PTZ-treated rats. A greater inhibitory effect was observed with CAR (3 mg/kg), which further decreased the levels of these inflammatory mediators by approximately 78%, 54%, 74%, and 62%, respectively. Notably, CAR (10 mg/kg) produced the most pronounced anti-inflammatory effect, markedly reducing hippocampal NLRP3, TNF-α, NF-κB, and IL-1β levels by approximately 93.1%, 70.8%, 89%, and 77.2%, respectively, compared with the PTZ group. Furthermore, the anti-inflammatory efficacy of CAR (10 mg/kg) was comparable to that observed in the VAL-treated group. One-way ANOVA revealed significant differences among groups for all measured inflammatory markers (*p* < 0.0001), with large effect sizes (η² = 0.990 for NLRP3, η² = 0.973 for TNF-α, η² = 0.981 for NF-κB, and η² = 0.988 for IL-1β), indicating a substantial magnitude of group differences in hippocampal inflammatory responses **(**Fig. 7**)**.

### Effect of CAR and VAL on hippocampal levels of PI3K and mTOR in PTZ-induced kindling model

**Fig. 8:**
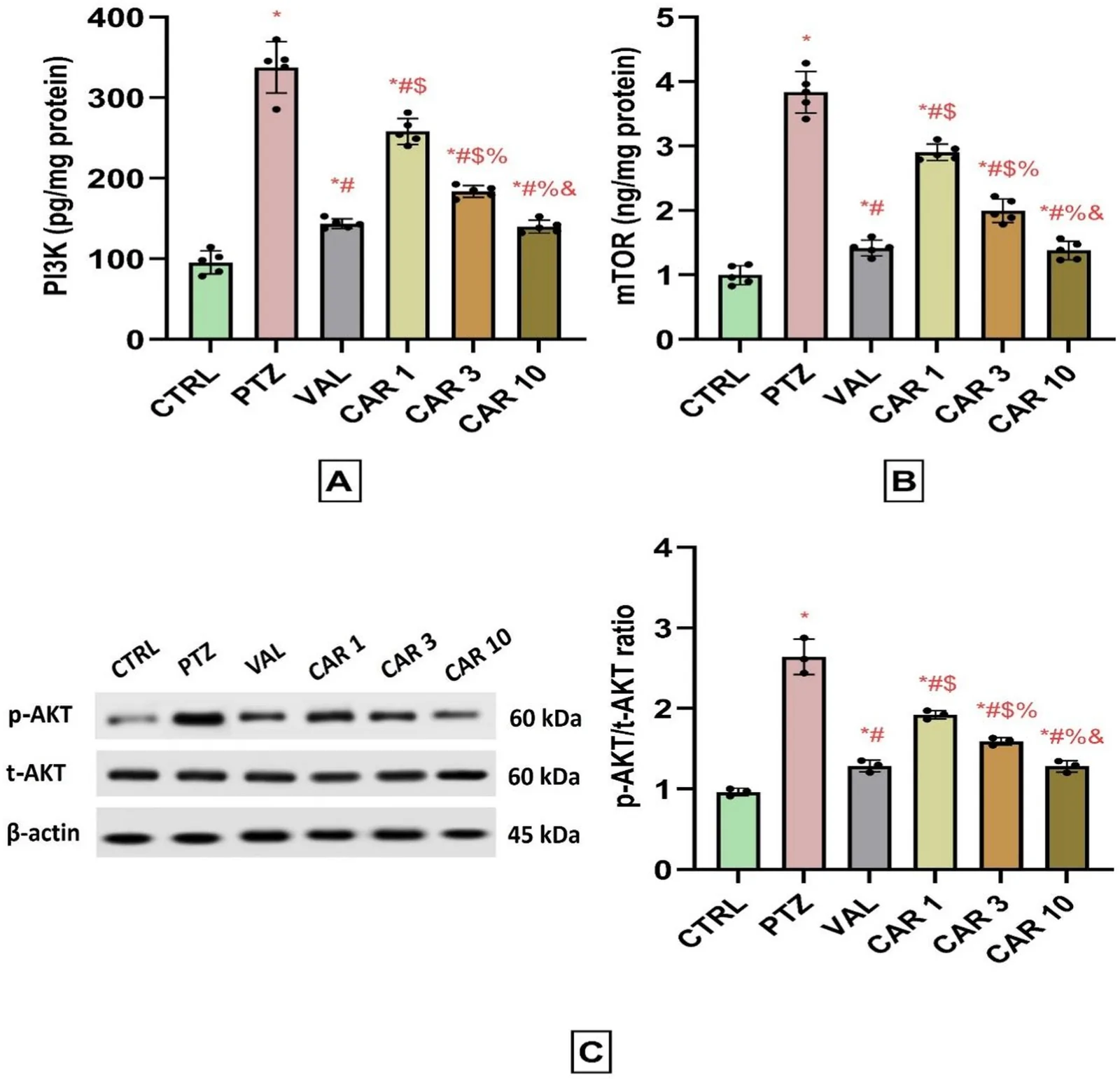
**Effect of CAR and VAL on hippocampal levels of PI3K**,** mTOR**,** and expression of AKT in PTZ-induced kindling model (A); PI3K (B); mTOR (C); phospho-AKT/total AKT ratio determined by western blot analysis**: Data are presented as mean ± SD. Statistical analysis was performed using one-way ANOVA followed by Tukey–Kramer’s multiple comparisons test (*n* = 5/group for PI3K and mTOR; *n* = 3/group for western blot analysis). *p*-values and effect sizes (η²) are reported. * Significant Vs CTRL, # Significant Vs PTZ, $ Significant Vs VAL 200, % Significant Vs CAR 1 and & Significant Vs CAR 3 (*p*˂0.05).

PTZ-induced kindling markedly activated the hippocampal PI3K/mTOR pathway, as reflected by significant increases in both PI3K and mTOR levels by approximately 3.5-fold and 3.8-fold, respectively, compared with the CTRL group. Treatment with VAL (200 mg/kg) significantly reduced both PI3K and mTOR levels by approximately 57% and 63%, respectively, compared with the PTZ group. CAR (1 mg/kg) reduced both PI3K and mTOR levels by approximately 23% and 24%, respectively, relative to PTZ-treated rats. A greater inhibitory effect was observed with CAR (3 mg/kg), which further decreased the levels of these mediators by approximately 45% and 48%, respectively. Notably, CAR (10 mg/kg) produced the most pronounced effect, markedly reducing hippocampal PI3K and mTOR levels by approximately 58% and 64%, respectively, compared with the PTZ group. Among the tested doses, CAR 10 mg/kg produced the greatest suppression of PI3K/mTOR signaling and achieved an effect comparable to that of VAL treatment. One-way ANOVA revealed significant differences among groups for both PI3K and mTOR levels (*p* < 0.0001), with large effect sizes (η² = 0.968 for PI3K and η² = 0.972 for mTOR), indicating a substantial magnitude of group differences in hippocampal PI3K/mTOR signaling-related markers **(**Fig. 8A**&B)**.

### Effect of CAR and VAL on hippocampal AKT phosphorylation status by western blot in PTZ-induced kindling model

Phosphorylation of AKT was markedly increased in the PTZ group, as evidenced by a 2.7-fold increment in the ratio of phospho-AKT to total AKT compared to the CTRL group. VAL (200 mg/kg, I.P.) significantly decreased phospho-AKT expression by approximately 51% relative to the PTZ group. Comparably, CAR (1 mg/kg, orally) decreased hippocampal phospho-AKT levels by about 27%, while a higher dose of CAR (3 mg/kg) induced a more pronounced decrease of approximately 39.6% compared to PTZ. Notably, the CAR (10 mg/kg) group produced the most substantial suppression of phospho-AKT expression by 51.5% compared to the PTZ group. One-way ANOVA revealed significant differences among groups in the phospho-AKT/total AKT ratio (*p* < 0.0001), with a large effect size (η² = 0.976), indicating a substantial magnitude of group differences in AKT phosphorylation status **(**Fig. 8C**)**.

## Discussion

The present study investigated the potential modulatory effects of CAR on epileptogenesis using a PTZ-induced kindling model. Epilepsy is increasingly conceptualized as a progressive network disorder arising from the interaction of numerous biological systems rather than isolated alterations in neuronal excitability. In this milieu, the progression of seizure susceptibility and epileptogenic remodeling are associated with oxidative stress, neuroinflammation, and synaptic reorganization^44,45^. Nevertheless, translating this conceptual progress into pharmacological interventions has proven challenging. Consequently, targeting convergent stress-associated pathways requires the development of pharmacological strategies that go beyond traditional ion channel modulation.

Even though it is not a noticeable candidate at first glance, CAR is of interest in this context. Its pharmacological profile, including dual β/α1-adrenergic blockade, along with preclinical data suggesting that CAR may moderate oxidant/antioxidant imbalance and linked pathways in neuronal systems^46,47^. Nevertheless, the extent to which these effects can modulate epileptogenic processes remains indeterminate. Most existing mechanistic insights originated from non-epileptic injury models, which restrict straightforward extrapolation to seizure-driven network alterations and provide provisional rather than established translational relevance.

In the existing work, the progressive escalation of seizure activity was observed through repeated PTZ administrations, demonstrating the development of seizure sensitization that are consistent with the broader idea of kindling initially recognized in electrical stimulation models^48^, and later extended to chemical models of epileptogenesis^49,50^. In the current analysis, CAR administration attenuated this progressive escalation in Racine scores, suggesting a modulatory action on the development of seizure susceptibility rather than an acute anticonvulsant effect. This dissimilarity is essential, as kindling models are considered to imitate features of epileptogenic development rather than transient seizure suppression^44,50^. However, in the absence of electrophysiological recordings, the exact impact of CAR on neuronal firing dynamics and network synchronization cannot be definitively determined.

Behaviorally, PTZ-kindled rats displayed lessened exploratory activity and a distinct shift toward anxiety-like behavior in the OFT. This is consistent with previously reported behavioral impairments in PTZ-induced kindling models^51,52^. These alterations have been construed as part of a wider neurobehavioral phenotype related to epileptogenic development instead of simple seizure expression^50^. The OFT is commonly used to measure locomotor and anxiety-linked behavior in rodents, even though its analysis should ideally be interpreted alongside complementary behavioral paradigms^53^. CAR partly improved these behavioral modifications. This indicated a reduced seizure burden and possible adrenergic modulation provided independent changes in emotional and exploratory behavior. Thus, behavioral recovery should be clarified carefully, as it likely reflects concurrent neurophysiological and pharmacological impacts instead of a solitary mechanistic pathway.

A marked neuronal loss and structural disorganization within the hippocampal CA3 subfield following PTZ exposure were illustrated by histopathological analysis. This is in line with the known susceptibility to excitotoxic damage following PTZ exposure due to its recurrent excitatory activity and high metabolic demand^37,54^. CAR administration partially preserved hippocampal architecture, suggesting mitigation of seizure-related neuronal damage.

A mechanistic perspective involved many interrelated mechanisms that contributed to the detected deleterious effects of PTZ-provoked seizures. PTZ primarily mediated seizures via inhibition of GABA_A receptor function, causing lessened inhibitory tone and enhanced neuronal excitability^55^. Existing work revealed that alterations in GABA levels after CAR administration may indicate a probable involvement in restoring inhibitory/excitatory balance.

Neuroinflammation represents a key mechanistic key in epileptogenesis. Activation of NF-κB signaling enhances transcription of pro-inflammatory cytokines, e.g., IL-1β and TNF-α, which promote neuronal excitability and facilitate seizure spread within hyperexcitable networks^10,56^. In the present study, PTZ exposure was associated with increased expression of NF-κB and downstream cytokines, whereas CAR treatment was linked to attenuation of these inflammatory markers.

Downstream of NF-κB signaling, the NLRP3 inflammasome acts as a key amplification platform linking inflammatory priming to cytokine maturation. Its activation requires NF-κB–dependent transcriptional priming followed by assembly of the inflammasome complex and caspase-1 activation, leading to the production of IL-1β. In the present study, CAR treatment was associated with reduced hippocampal levels of NF-κB, NLRP3, IL-1β, and TNF-α, indicating reduced expression of inflammatory markers related to the NF-κB/NLRP3 axis.

The anti-inflammatory effects observed in the present study are supported by previous experimental evidence demonstrating that CAR modulates multiple components of inflammatory signaling cascades. Amirshahrokhi and Abzirakan^25^ reported that CAR significantly reduced the expression of pro-inflammatory mediators, including TNF-α and IL-1β, while suppressing NF-κB activation and attenuating oxidative stress in an experimental model of type 1 diabetes. Moreover, Wong and Li^57^ demonstrated that CAR directly inhibited NLRP3 inflammasome activation, leading to reduced production of IL-1β and other downstream inflammatory mediators. Collectively, these observations indicate that CAR treatment was associated with concurrent changes in oxidative stress and inflammatory markers. However, the present design cannot determine whether these changes reflect direct pathway modulation or secondary consequences of reduced behavioral seizure severity.

Oxidative stress is progressively distinguished as a crucial and critical issue in epileptogenesis, acting at the interface between metabolic dysfunction, mitochondrial instability, and neuroinflammatory activation^58,59^. In neuronal systems, excessive production of reactive oxygen species (ROS) interrupts cellular redox homeostasis, causing lipid peroxidation, protein oxidation, and diminished mitochondrial function, contributing to enhanced neuronal excitability and reduced seizure threshold^14,58^.

ROS at the molecular level is not just a result of cellular stress but acts as an active signaling mediator. Experimental evidence indicates that oxidative stress can accelerate the activation of redox-sensitive transcription factors, particularly NF-κB, thereby promoting the transcription of pro-inflammatory cytokines and amplifying neuroimmune responses^60,61^. Moreover, NF-κB–dependent signaling can also increase oxidative burden via production of pro-oxidant enzymes, creating a bidirectional feed-forward loop that sustains both oxidative and inflammatory cascades within neurons^62^. This reciprocal relationship provides a potential mechanistic basis for the progressive nature of seizure-provoked neuronal damage.

In this outline, oxidative stress is also closely related to inflammasome activation. Increased ROS production has been implicated in priming and activating the NLRP3 inflammasome, thus assisting caspase-1–dependent IL-1β maturation and strengthening neuroinflammatory signaling pathways^56,63^. Such relationships suggest that oxidative stress may represent an important, interconnected process linking metabolic dysfunction and inflammatory responses within epileptogenic networks.

Current outcomes revealed that PTZ-kindled rats exhibited a marked alteration in oxidative status demonstrated by increased MDA and suppression of TAC, showing heightened lipid peroxidation and depletion of endogenous antioxidant defenses. These results are consistent with preceding seizure- provoked brain damage in animal models, where oxidative imbalance has been repeatedly established as a basic pathological characteristic^58,64^. In contrast, CAR administration restored redox balance, as demonstrated by decreased MDA and restored TAC levels. Such antioxidant outcomes of CAR have been recognized in numerous experimental injury models, as shown to reduce ROS production, inhibit lipid peroxidation, and preserve mitochondrial integrity under oxidative stress conditions^47,65^. These actions are mechanistically related to the epileptogenesis context, as mitochondrial dysfunction and oxidative damage are closely related to neuronal hyperexcitability and seizure propagation.

Alterations in PI3K/AKT/mTOR signaling have been increasingly recognized as contributors to epileptogenesis, synaptic remodeling, and activity-dependent structural plasticity^17^. However, the biological role of this signaling pathway is highly context dependent. In the central nervous system, PI3K/AKT/mTOR signaling is essential for neuronal survival, protein synthesis, synaptic plasticity, and metabolic homeostasis under physiological conditions, whereas its dysregulation has been associated with several neurological disorders, including epilepsy^17,18,66^. Therefore, the present findings should not be interpreted as indicating that suppression of PI3K/AKT/mTOR signaling is universally beneficial. Rather, they demonstrate treatment-associated alterations in PI3K/AKT/mTOR-related measurements within the specific context of the PTZ window-kindling model. Notably, emerging evidence indicates that PI3K/AKT/mTOR signaling does not operate in isolation but interacts extensively with oxidative stress and inflammatory pathways, forming a broader homeostatic network that may become dysregulated under chronic excitatory conditions^17,66^.

Experimental studies in neurological injury and metabolic stress models have demonstrated that dysregulation of PI3K/AKT/mTOR signaling is frequently associated with both inflammatory amplification and redox imbalance, where ROS and cytokine-mediated signaling pathways converge to modulate downstream mTOR activity and synaptic remodeling processes^17,19,67^.

In the present study, PTZ-induced kindling was associated with alterations in PI3K, AKT, and mTOR expression, indicating changes in these signaling components under epileptogenic stress conditions. CAR administration was associated with alterations in PI3K/AKT/mTOR-related measurements. These observations are broadly consistent with previous studies, as Niapour and Hosseinzadeh^68^ reported that CAR altered p-AKT and PI3K-related signaling responses in paclitaxel-resistant gastric cancer cells. Moreover, Mahmoud et al.^69^ reported that CAR counteracted the renal damage induced by doxorubicin via suppressing oxidative stress, inflammatory response, and apoptosis through downregulation of PI3K/AKT/mTOR signaling.

Collectively, the findings indicate that CAR treatment was associated with improvements across multiple epileptogenesis-related domains, including seizure progression, neuronal integrity, neurotransmitter balance, oxidative status, and inflammatory markers. These findings support an integrative interpretation of CAR-associated effects involving interconnected biological processes rather than a single pathway-specific mechanism.

The contribution of the present study should be interpreted as integrative rather than target-discovery based. The antioxidant and anti-inflammatory properties of CAR and the involvement of NLRP3-related inflammatory processes and PI3K/AKT/mTOR signaling in experimental epilepsy have been described previously. The present findings extend these observations by characterizing, within a single PTZ window-kindling experiment, concurrent treatment-associated changes across behavioral, histopathological, neurochemical, oxidative, inflammatory, and pathway-related endpoints. However, this integrated assessment does not establish a novel molecular target, demonstrate functional interactions among these processes, or determine whether the molecular alterations are causes or consequences of the lower behavioral seizure scores.

Although these findings provide an integrated characterization of CAR-associated changes during experimental kindling, caution should be exercised when extrapolating the present results to human epilepsy because of the inherent limitations of chemically induced seizure models.

The translational relevance of the present findings should be interpreted cautiously. The PTZ window-kindling model is useful for investigating progressive behavioral seizure susceptibility and associated molecular alterations, but it does not reproduce the spontaneous recurrent seizures, chronic disease course, diverse etiologies, or electroclinical heterogeneity of human epilepsy. Moreover, PTZ-induced kindling predominantly models chemically induced generalized seizure progression and cannot represent the full spectrum of focal and generalized epilepsy syndromes. Therefore, the present results should be considered specific to this experimental paradigm and require confirmation in complementary models incorporating spontaneous seizures, chronic EEG monitoring, and post-establishment therapeutic intervention.

## Conclusion

In conclusion, CAR treatment was associated with attenuation of behavioral seizure progression and concurrent improvements in open-field performance, hippocampal histopathology, GABA content, oxidative status, inflammatory mediators, and alterations in PI3K/AKT/mTOR-related measurements in the PTZ window-kindling model. The contribution of this study lies in providing an integrated characterization of these treatment-associated changes within a single experimental paradigm rather than identifying a novel molecular target or establishing a pathway-specific mechanism. Since the current study did not include EEG monitoring, functional pathway interventions, canonical assessments of NLRP3 inflammasome activation, or post-kindling treatment paradigms, these findings should not be interpreted as evidence of direct mechanistic causality, definitive disease modification, or therapeutic efficacy in established epilepsy. Further studies incorporating chronic EEG monitoring, pathway-specific approaches, post-kindling treatment designs, and complementary models of focal and spontaneous recurrent seizures are warranted to determine the mechanistic and translational relevance of CAR.

### Limitation

Several limitations should be acknowledged. First, although CAR treatment was associated with modulation of NLRP3 inflammasome- and PI3K/AKT/mTOR-related proteins, no pathway-specific inhibitors, genetic approaches, or rescue experiments were performed. Therefore, these findings indicate an association rather than a definitive causal mechanism. Second, although the modified PTZ window-kindling model is well established for studying epileptogenesis, it does not fully recapitulate the complexity or spontaneous seizure activity of human epilepsy, limiting direct clinical translation. Third, seizure progression was assessed using the modified Racine scale without concurrent EEG monitoring. While this approach is widely accepted in PTZ-kindling studies, it cannot verify electrographic seizure activity or distinguish suppression of seizure expression from true modification of epileptogenesis. Fourth, behavioral evaluation was limited to seizure scoring and the open-field test and therefore did not comprehensively assess epilepsy-associated cognitive and affective impairments. Finally, the relatively small number of biological replicates used for certain molecular analyses, particularly Western blot experiments (*n* = 3/group), warrants cautious interpretation of these findings. Accordingly, further studies incorporating mechanistic validation, continuous video-EEG monitoring, comprehensive behavioral assessments, and larger independent cohorts are required to strengthen and extend the present findings.

## Supplementary Information

Below is the link to the electronic supplementary material.

Supplementary Material 1

## Data Availability

The datasets used and/or analyzed during the current study are available from the corresponding author on reasonable request.
