# Supplementary material for: Carvedilol attenuates PTZ-induced epileptogenesis: associations with hippocampal neuroinflammation and PI3K/AKT/mTOR-related alterations
Source: Sci Rep. 2026 Aug 25;16:26640. doi: 10.1038/s41598-026-67306-2 (PMC13507096; doi:10.1038/s41598-026-67306-2)
Supplement: Supplementary file 1 [file 41598_2026_67306_MOESM1_ESM.pdf]

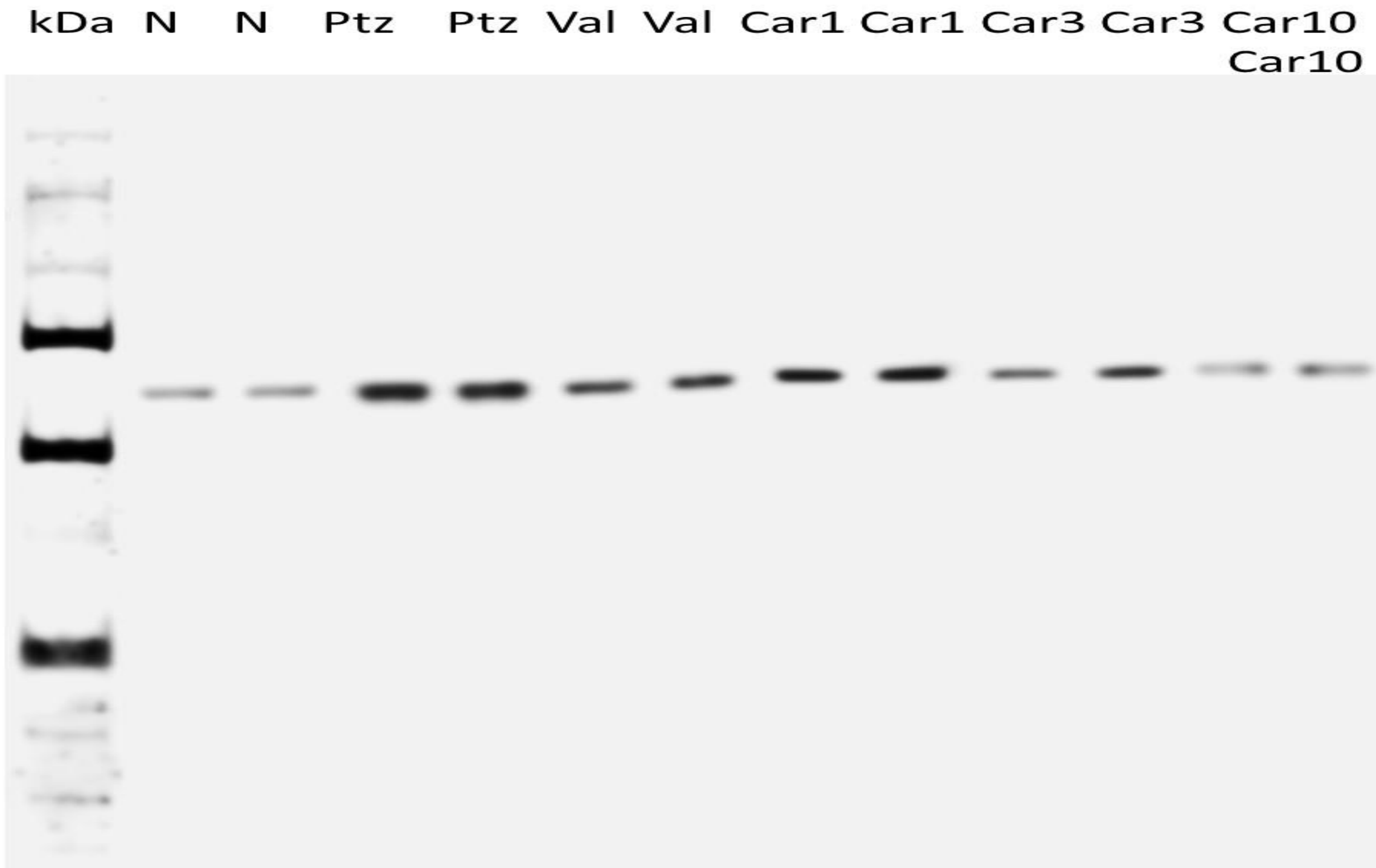
kDa N N Ptz Ptz Val Val Car1 Car1 Car3 Car3 Car10
Car10

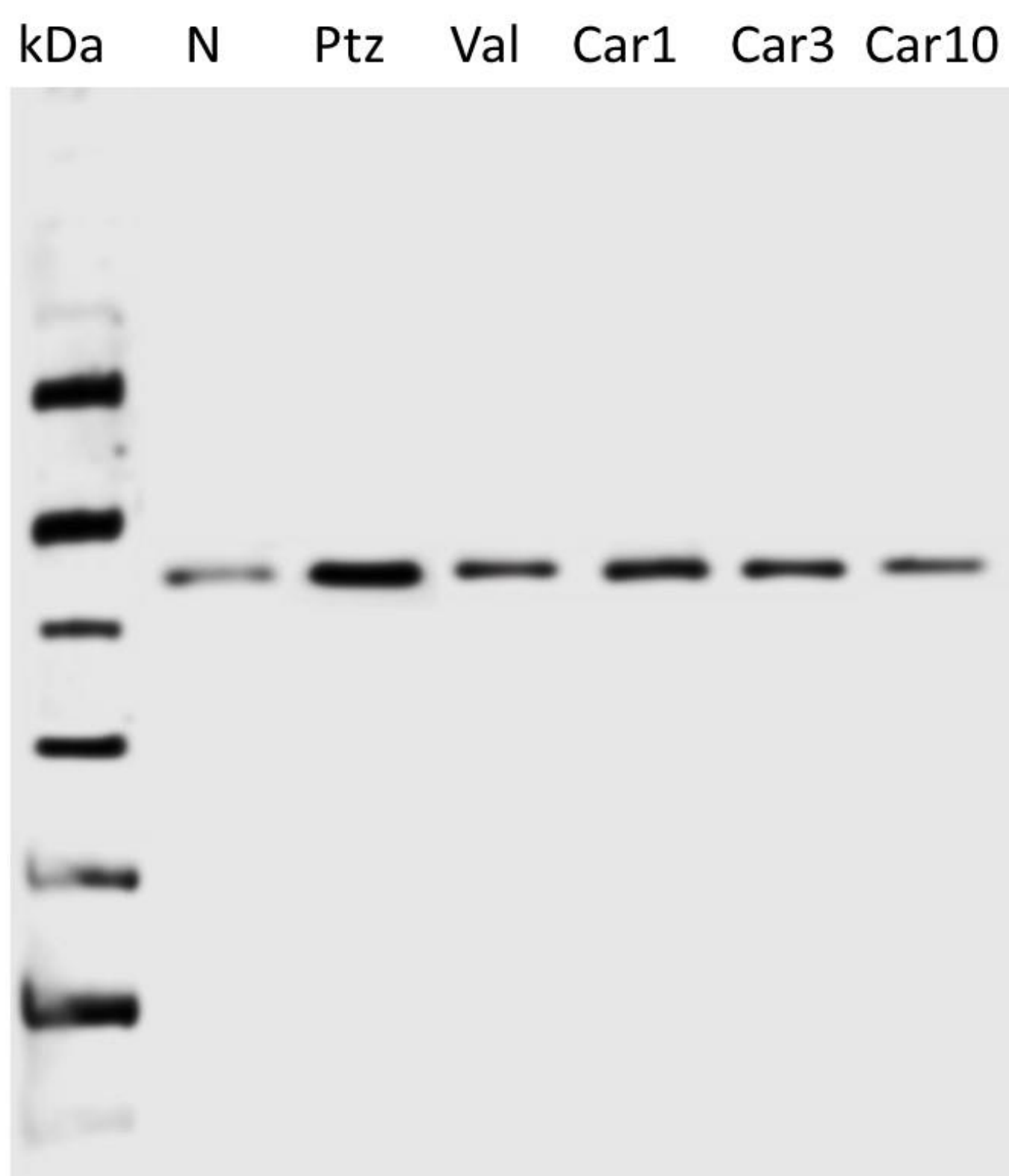
kDa N Ptz Val Car1 Car3 Car10

p-AKT

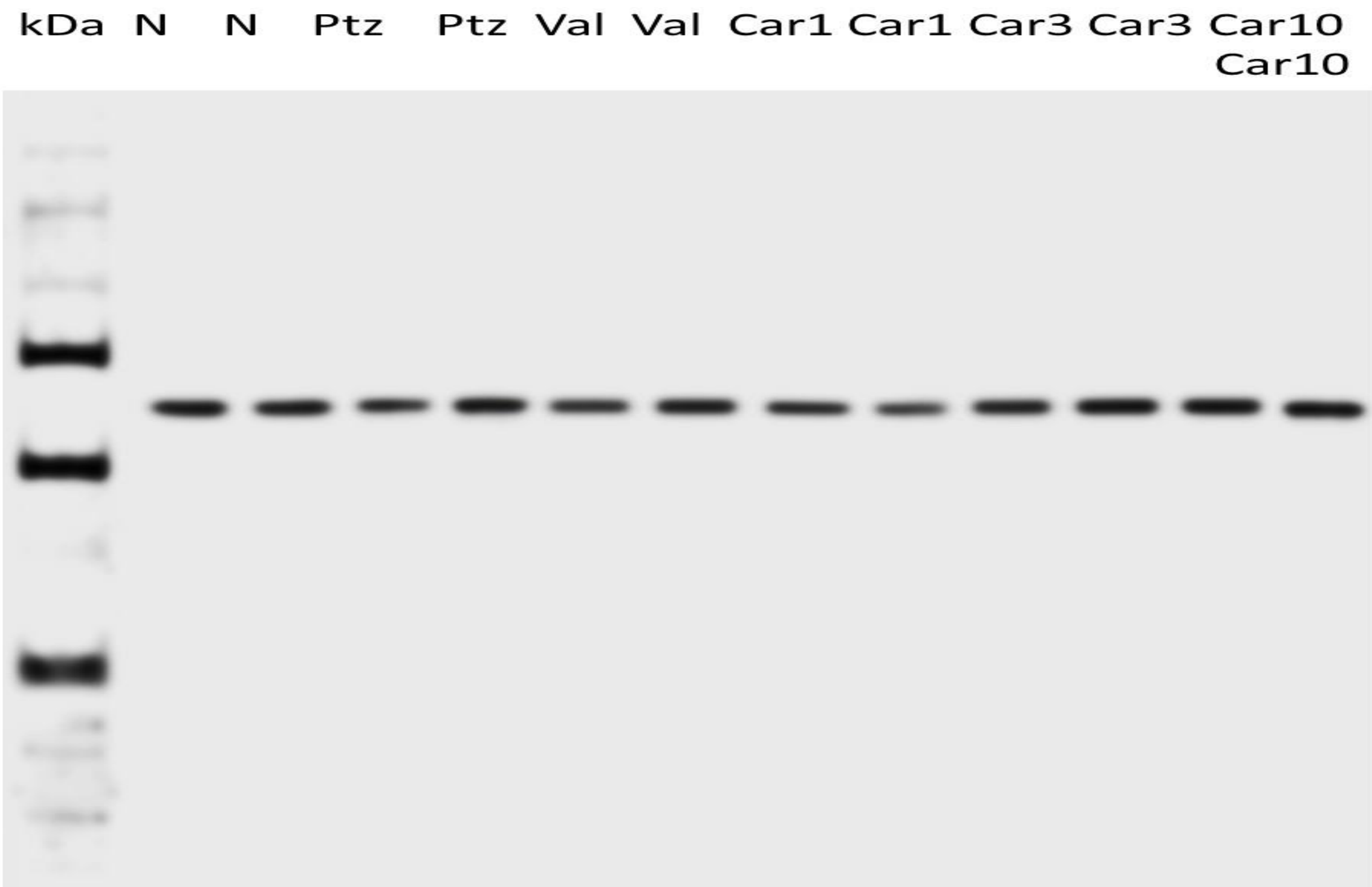
kDa N N Ptz Ptz Val Val Car1 Car1 Car3 Car3 Car10 Car10

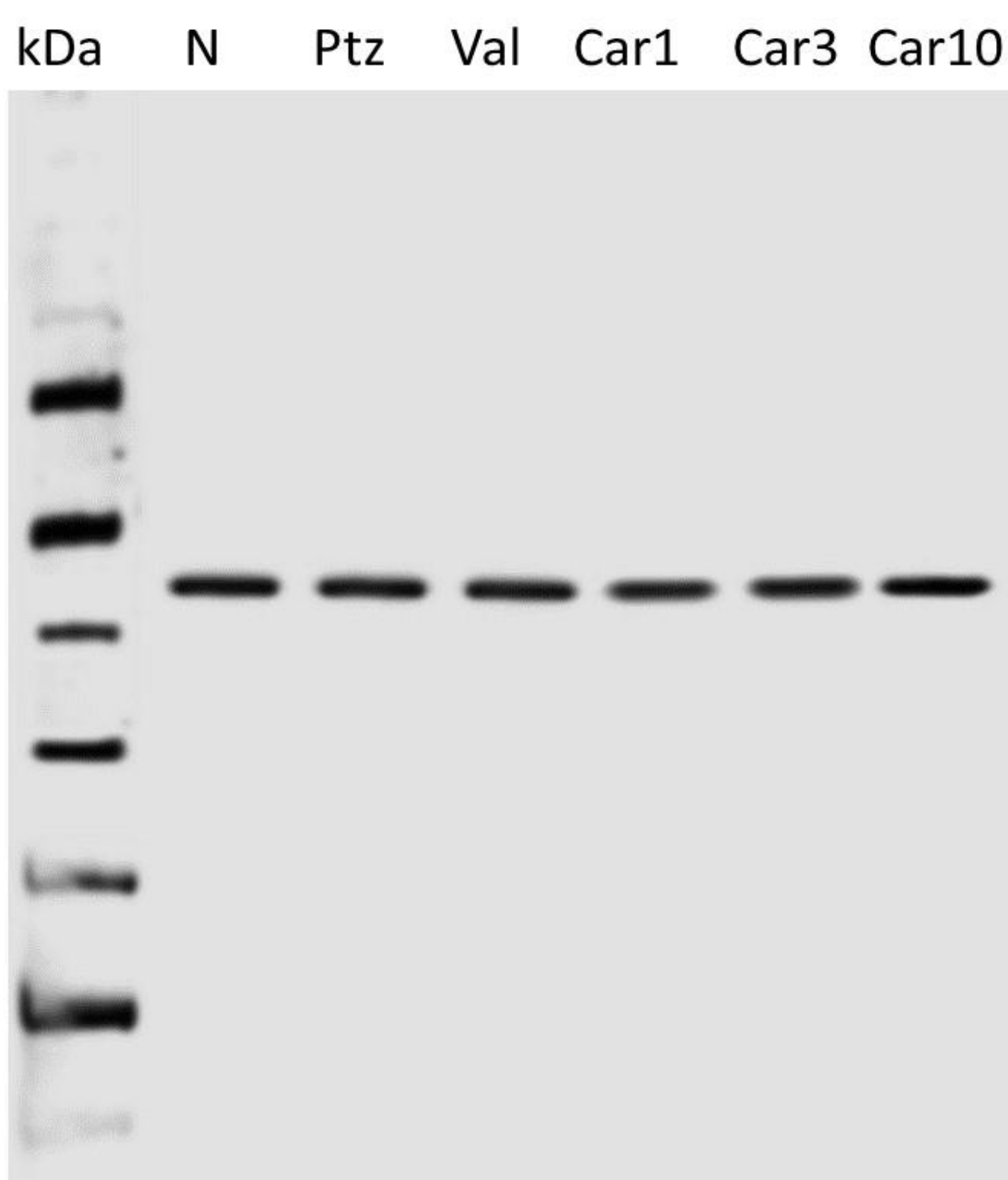
kDa N Ptz Val Car1 Car3 Car10

t-AKT

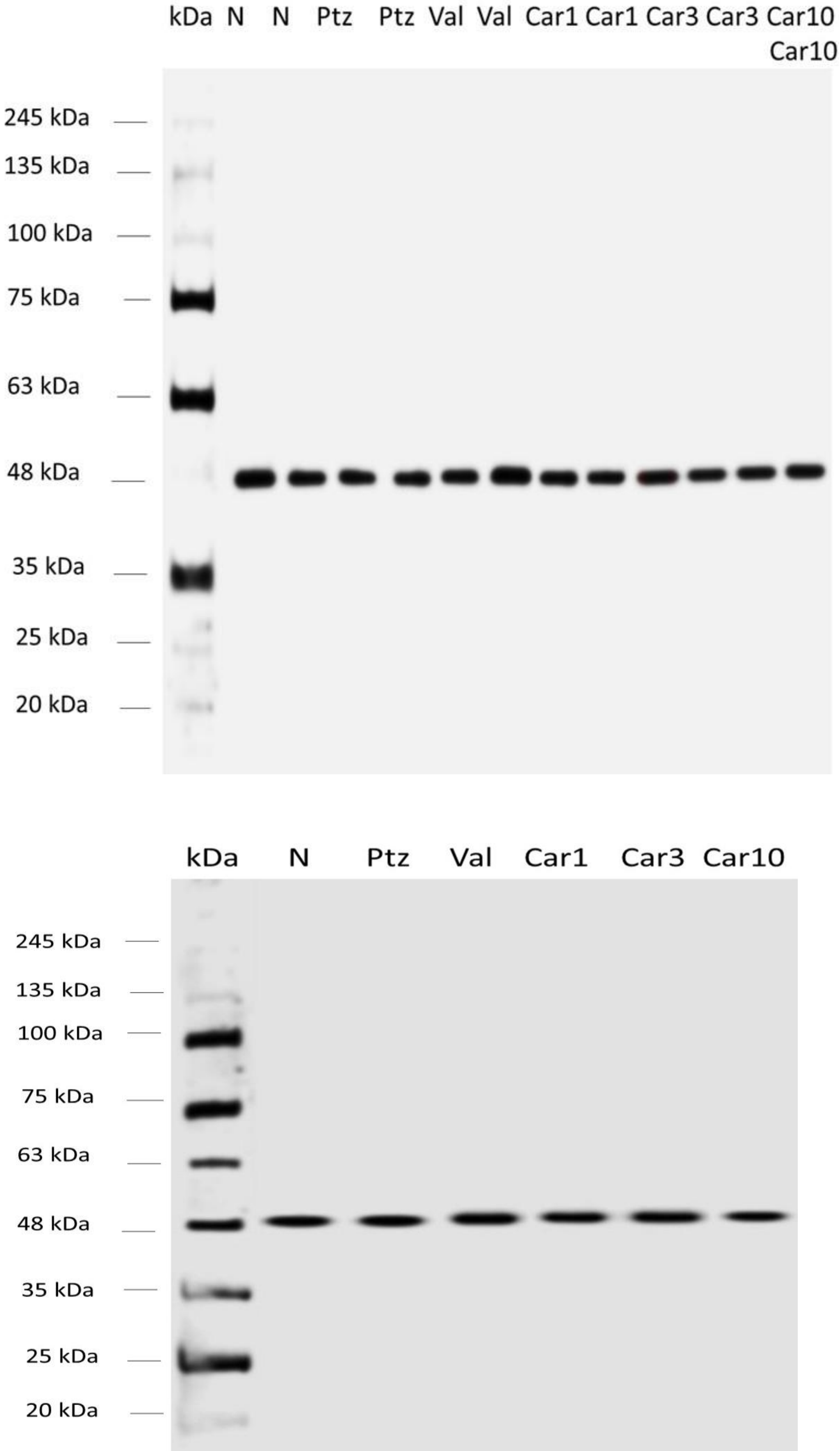
kDa N N Ptz Ptz Val Val Car1 Car1 Car3 Car3 Car10
Car10
245 kDa
135 kDa
100 kDa
75 kDa
63 kDa
48 kDa
35 kDa
25 kDa
20 kDa
kDa N Ptz Val Car1 Car3 Car10
245 kDa
135 kDa
100 kDa
75 kDa
63 kDa
48 kDa
35 kDa
25 kDa
20 kDa

Beta actin

Supplementary Table S1. Characteristics of the ELISA assay kits used in the study including assay format, standard calibration concentrations, detection range, and absorbance wavelength.

Biomarker	ELISA kit (Manufacturer, Cat. No.)	Assay format	Standard concentrations	Detection range	Absorbance
**GABA**	FineTest (ER1707)	Competitive ELISA	0, 31.25, 62.5, 125, 250, 500, 1000, 2000 pg/mL	31.25–2000 pg/mL	450 nm
**MDA**	MyBioSource (MBS268427)	Sandwich ELISA	0, 0.156, 0.312, 0.625, 1.25, 2.5, 5 nmol/mL	0.156–10 nmol/mL	450 nm
**TAC**	AMSBIO (AMS.E02T0028)	Competitive ELISA	0, 0.5, 1.0, 2.5, 5.0, 10.0 ng/mL	0.5-10 ng/mL	450 nm
**NLRP3**	MyBioSource (MBS2033695)	Sandwich ELISA	0, 0.312, 0.625, 1.25, 2.5, 5, 10, 20 ng/mL	0.312-20 ng/mL	450 nm
**TNF-α**	BioLegend (438204)	Sandwich ELISA	0, 15.6, 31.3, 62.5, 125, 250, 500, 1000 pg/mL	15.6–1000 pg/mL	450 nm
**NF-κB**	CUSABIO (CSB-E08788r)	Sandwich ELISA	0, 1.56, 3.125, 6.25, 12.5, 25, 50, 100 pg/mL	1.56–100 pg/mL	450 nm
**IL-1β**	R&D Systems (RLB00)	Sandwich ELISA	0, 31.3, 62.5, 125, 250, 500, 1000, 2000 pg/mL	31.3–2000 pg/mL	450 nm
**PI3K**	FineTest (ER1910)	Sandwich ELISA	0, 31.25, 62.5, 125, 250, 500, 1000, 2000 pg/mL	31.25–2000 pg/mL	450 nm
**mTOR**	FineTest (ER1520)	Sandwich ELISA	0, 0.156, 0.312, 0.625, 1.25, 2.5, 5, 10 ng/mL	0.156–10 ng/mL	450 nm
